# Long non-coding RNA H19 regulates proliferation and doxorubicin resistance in MCF-7 cells by targeting PARP1

**DOI:** 10.1080/21655979.2020.1761512

**Published:** 2020-05-13

**Authors:** Yu Wang, Peihong Zhou, Ping Li, Fengxia Yang, Xue-qiang Gao

**Affiliations:** a Department of Breast Surgery, The Affiliated Hospital of Qingdao University, Qingdao, Shandong, China; b Department of Operation Room, The Affiliated Hospital of Qingdao University, Qingdao, Shandong, China; c Department of Ultrasound, The Affiliated Hospital of Qingdao University, Qingdao, Shandong, China

**Keywords:** Breast cancer, chemotherapy, doxorubicin, lncRNA H19, PARP1

## Abstract

Chemoresistance is a major obstacle to effective breast cancer chemotherapy. However, the underlying molecular mechanisms remain unclear. The long noncoding RNA H19 (H19) is involved in various stages of tumorigenesis, however, its role in doxorubicin resistance remains unknown. The goal of this study was to evaluate the role of H19 in the development of doxorubicin-resistant breast cancer. Quantitative real-time PCR (qRT-PCR) analyzed H19 expression in chemotherapy-resistant and sensitive breast cancer tissues. Both knockdown and overexpression of H19 were used to assess the sensitivity to doxorubicin in breast cancer cells *in vitro and in vivo*. qRT-PCR and Western blot were used to explore the doxorubicin resistance mechanism of H19. We observed that the H19 expression was significantly upregulated in chemotherapy-resistant breast cancer tissues and doxorubicin-resistant breast cancer cell lines. Knockdown of H19 enhanced the sensitivity to doxorubicin both *in vitro* and *in vivo*. While H19 overexpression developed doxorubicin-resistant in breast cancer cells both *in vitro* and *in vivo*. Furthermore, it was revealed that H19 negatively regulated PARP1 expression in breast cancer cells following doxorubicin treatment. Knockdown of H19 sensitized breast cancer cells to doxorubicin by promoting PARP1 upregulation. H19 overexpression could recapitulate doxorubicin resistance by PARP1 downregulation. Our findings revealed that H19 plays a leading role in breast cancer chemoresistance development, mediated mainly through a H19-PARP1 pathway.

## Graphical Abstract

**Figure:**
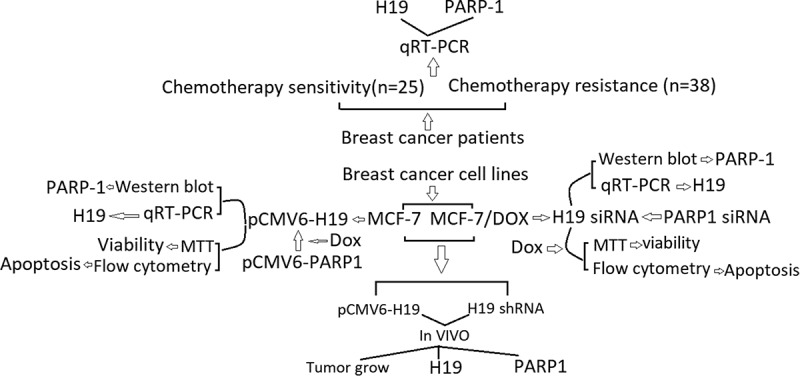

## Introduction

Breast cancer was regarded as the second leading cause of death caused by cancer among women after lung cancer [1]. Currently, chemotherapy is the treatment of choice for breast cancer which initiates tumor cell apoptosis while other therapeutic modalities highlight works on hormone receptor resistance and targeted therapy. After receiving treatment, patients with breast cancer may have long-term survival with almost 90% of them showing a 5-year survival rate [2]. However, intrinsic or acquired drug resistance is a major impediment to the successful treatment of women with breast cancer using chemotherapy. With the progress of treatment, patients often become resistant to chemotherapy and result in treatment failure [3,4]. Therefore, it is necessary to explore the molecular mechanisms that mediate breast cancer formation and chemotherapy resistance that is useful in treating the disease.

Long noncoding RNAs (lncRNAs) have recently gained considerable attention as key players in biological regulation; however, the mechanisms by which lncRNAs govern various disease processes remain mysterious and are just beginning to be understood [5]. Some lncRNAs have been proved to play an important role in the tumor drug resistance [6], such as lncRNA MIR100HG which mediates cetuximab resistance via Wnt/beta-catenin signaling [7]. lncRNA HULC triggers autophagy attenuates the chemosensitivity of HCC cells [8]. A large number of clinical studies have suggested that long noncoding RNA H19 (H19) can serve as a potential biomarker for the diagnosis of breast cancer. High expression levels of H19 increase the drug resistance of breast cancer cells and is associated with poor prognosis within patients with breast cancer [9]. For example, circulating H19 has been proved to predict presurgical response to neoadjuvant chemotherapy in breast cancer, high H19 levels had poor response to neoadjuvant chemotherapy [10]. H19 overexpression was negatively correlated to the trastuzumab-therapy response, and H19 downregulation could reverse trastuzumab resistance and enhance the inhibitory function of this drug [11]. H19 overexpression was observed in doxorubicin-resistant breast cancer cells, and H19 downexpression significantly decreased doxorubicin resistance by reducing cell viability and inducing apoptosis [12]. However, the biological mechanism of H19 in regulating doxorubicin sensitivity in breast cancer has not been fully elucidated. Si et al. [13] reported that H19 attenuated cell apoptosis in response to PTX treatment by inhibiting transcription of pro-apoptotic genes BIK and NOXA. Zhu et al. [14] reported that H19 mediated breast cancer chemoresistance mainly through a H19-CUL4A-ABCB1/MDR1 pathway. Han et al. [15] reported that targeting H19 might restore chemo-sensitivity in paclitaxel-resistant breast cancer by mediating the AKT signaling pathway. However, the biological role of H19 in predicting postsurgical chemotherapy response in patients with breast cancer and on doxorubicin chemoresistance in breast cancer cells remains poorly understood.

Poly (ADP-ribose) polymerase (PARP) catalyzes the attachment of ADP-ribose units to itself and its target proteins to recruit the DNA repair machinery to the site of DNA damage. PARP1 also functions in other cellular processes, including transcriptional regulation and cell cycles [16]. Recent clinical data confirmed that the cytoplasmic PARP-1 expression in the needle core biopsies of breast cancer was low, and low PARP-1 expression was associated with no or poor response to chemotherapy [17]. Haynes et al. [18] reported that PARP-1 stabilization/hyperactivation induced cell death and reversed chemo-resistance in breast cancer cells. Thus, we speculated that PARP1 is involved in chemotherapy response in breast cancer.

Previous studies demonstrated that loss of some long non-coding RN (lncRNA) leads to PARP1 up-regulation in cells or tissues, such as lncRNA MALAT1 [19], lncRNA Mhrt [20], lncRNA GAS5 [21] and lncRNA FOXD3-AS1 [22]. However, whether H19 takes part in the dysregulation of PARP1 expression in breast cancer cells remains unknown.

In the present study, we determined the importance of H19 in breast cancer chemoresistance using doxorubicin as a model chemotherapeutic agent **in vitro and in vivo.**

## Materials and methods

### Patients samples

Breast cancer samples were collected from Department of Breast surgery, the affiliated Hospital of Qingdao University (Qingdao, China) between 2015 and 2016. A total of 63 pairs of breast cancer tissues and adjacent normal tissues were examined in the study. All patients underwent conventional doxorubicin (Dox)-based chemotherapy. Patients with disease progression or recurrence 6 months or less after Dox therapy were defined as Dox resistance, whereas those without recurrence or recurrence 24 months after Dox therapy were defined as Dox sensitivity. After systemic Dox treatments, 25 Dox-sensitive patients contributed to 25 pairs of breast tumor tissues and 25 of normal tissues, and 38 Dox-resistant patients contributed 38 pairs of breast tumor and normal tissues. All of the samples were retrieved within 15 min after the surgery and immediately snap-frozen in liquid nitrogen and stored at −80°C until used for gene expression analysis. This study was approved by the Clinical Ethics Review Board at the affiliated hospital of Qingdao University and written informed consents were from all patients at their recruitment time.

### Cell lines and cell culture

The human breast cancer MCF-7 cells were purchased from Institute of Cell Research, Chinese Academy of Sciences (Beijing, China). Doxorubicin (Dox)-resistant MCF-7/Dox cells were established by induction with gradient concentrations of Dox *in vitro*. Cells were cultured in DMEM (Sigma) supplemented with 10% fetal bovine serum (Gibco), 100 units/ml of penicillin, 100 μ g/ml, streptomycin in a 5% CO_2_ atmosphere at 37°C. MCF-7/Dox cells were cultured at a final concentration of 1 μM.

### Construction of the H19 shRNA lentivirus

The lentivirus expressed short hairpin RNA (shRNA) targeting the H19 gene was constructed by GeneCopoeia (Shanghai, China), with the lentivirus-mediated scrambled shRNA generated as a negative control. The interfering sequences specifically targeting the H19 gene was 5′-CGTCATGTTCTGGTTTGAGAT-3′. The lentivirus particles were produced with a third-generation lentivirus packaging system. Briefly, three auxiliary plasmids (pRRE, pRSV-Rev, and pCMV-VSVG) and the core plasmid were transfected into human embryonic kidney 293 T cells (HEK293 T) using the Lipo6000 transfection reagent (Beyotime Biotechnology, Shanghai, China). The viral particles were extracted 72 h after transfection, and lentivirus particles were further concentrated. Six hundred sixty-one W cells were then infected with virus particles, and polybrene (Beyotime Biotechnology, Shanghai, China) was added to enhance infection efficiency. To avoid virulence, media were replaced with fresh complete DMEM media 24 h later. To obtain the cells that were consistently knocking down the target gene, cells were screened with hygromycin (Sangon Biotech, Shanghai, China) or puromycin (Beyotime Biotechnology, Shanghai, China) at an initial concentration of 8 μg/mL 2 days after infection of viral particles. Two weeks after screening, cells with stable H19 knockdowns were obtained. The stably transfected colonies were named Lv-H19 shRNA and Lv-NC shRNA.

### Plasmids transfection

H19 expression vector pCMV6-H19 (clone ID: 4993796), PARP-1 expression vector pCMV6- PARP-1 and the vector construct (pCMV6-Entry) was obtained from OriGene Technologies, Rockville, MD. Breast cancer cells were transfected with pCMV6-H19, pCMV6-PARP-1 and pCMV6 using Lipofectamine LTX (Life Technologies) following the manufacturer’s instructions. Briefly, cells in a 6-well plate were transfected with 1 µg plasmid using Lipofectamine LTX (Life Technologies). 48 h post-transfection, cells were trypsinized and plated in 10 cm dishes to establish stable clones. Colonies that survive after a 2-3-week selection in 400 µg/ml G418 were isolated under fluorescence microscope. Stable cells in six-well plates were transfected with 10 ng plasmid.

### siRNA transfection

siRNAs targeting human H19 (H19 siRNA), siRNAs targeting human PARP-1 (PARP-1 siRNA), and non-targeting siRNAs (NC siRNA) were obtained from Santa Cruz Biotechnology (Shanghai, China). For siRNA-mediated knockdown studies, cells were transfected with 500 pmol of siRNA specific for H19 or PARP-1 or NC siRNA. After 8 h, the transfection medium was replaced with DM. Subsequent assays were conducted 24 to 48 h after transfection.

### Drugs treatment

Doxorubicin was obtained from Sigma (Shanghai, China). MCF-7 and MCF-7/Dox cells were treated for 72 h with doxorubicin (0 to 120 μM). Cytotoxicity was measured using MTT. To observe the effect of H19 or PARP-1 on Dox-induced cytotoxicity, cells were treated with 1.2uΜDox for 72 h.

### Cellular viability assay

Cell viability was monitored by 2-(4, 5-dimethyltriazol-2-yl)-2,5-diphenyl tetrazolium bromide (MTT; Sigma) colorimetric assay. Briefly, 20 μL of MTT (5 mg/ml) was added to each well. After 4 hr of incubation at 37°C, cell supernatants were discarded. MTT crystals were dissolved with DMSO, while absorbance was measured at 570 nm. All experiments were done with 6–8 wells per experiment and repeated at least three times.

### Flow cytometry analysis of apoptosis

The apoptosis assay was performed using ApoTargetTM annexin V-FITC kit (BioSource International, Inc., Camarillo, CA, USA) and following the manufacturer’s protocol. At each indicated time point, the cells were harvested using Trypsin-EDTA (Sigma-Aldrich) and washed once in 1 × PBS. Then, the cell apoptosis was analyzed using Annexin V and propidium iodide (PI). Apoptotic cells were identified as Annexin V-FITC‐positive and PI-positive cells.

### qRT-PCR

Total RNA was isolated from cells or frozen tissue with the RNA extraction kit (Nanjing, China) according to the manufacturer’s instructions. RNA quality was measured using NanoDrop (Thermo Scientific). Reverse transcription reaction for H19 and PARP-1 was performed using a SuperScript® III First-Strand Synthesis System (Invitrogen). H19 expression levels were quantified by real-time qRT-PCR using miScript Primer assay (Qiagen) and normalized to RNU6. PARP-1 expression levels were normalized to GAPDH.

### Western blotting analysis

Whole-cell lysates were prepared using 2% SDS, sonicated, and centrifuged (12,000 rpm) at 4°C for 15 min. The supernatants were boiled for 5 min and size-fractionated by SDS-PAGE (7.5% acrylamide). After transferring proteins onto nitrocellulose filters, the blots were incubated with primary PARP-1 andβ-actin antibody following incubations with secondary antibody, immunocomplexes were developed by using chemiluminescence.

### Animal experiment

Female BALB/c nude mice (5–6 weeks of age, 16–18 g) were obtained from National Rodent Shanghai Experimental Branch Center, Chinese Academy of Sciences (Shanghai, China). All experimental procedures involving animals were conducted in accordance with the institutional guidelines by the Affiliated hospital of Qingdao University.

MCF-7/Lv-H19, MCF-7/Lv-NC, MCF-7/Dox/H19 shRNA and MCF-7/Dox/NC shRNA cells [0.2 mL (2 × 10^6^)] were subcutaneously injected into the right back of the mice, respectively. Once tumor volume grew to (4 weeks) about 100 cm^3^, the mice (*n* = 8 per group) were, respectively, treated with PBS (0.1 mL, tail i.v. injection), Dox (0.1 mL, 10 mg/kg, tail i.v. injection, 4 times/week) and housed for another 3 consecutive weeks. Tumor volume was measured once every 3 days and volumes were evaluated by the following formula: tumor volume = (length × width^2^)/2. All mice were killed by intraperitoneal injection of 200 mg/kg pentobarbital at the end of the experiment. The tumor specimens were carefully excised and stored at −80°C for further use.

### Statistical analyses

Statistical analyses were carried out by using SPSS 22.0 soft (IBM, SPSS, Chicago, IL, USA). The data are presented as the mean ± SD. Differences between groups were analyzed by one-way analysis of variance (ANOVA) and Student’s t-test. A p-value <0.05 was considered statistically significant.

## Results

### H19 overexpression in breast cancer tissue correlates with chemoresistance

**Figure 1.**
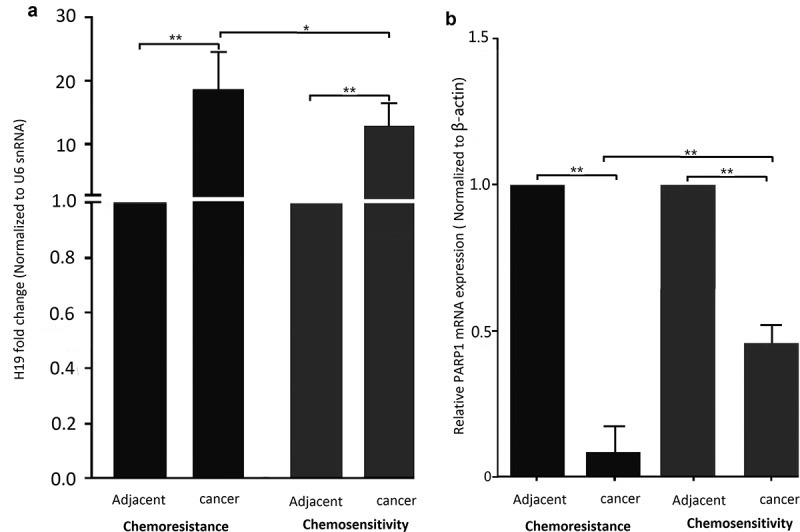
H19 contributes to chemoresistance in breast cancer tissues and cell lines. (a) qPCR analysis showed that H19 expression levels were higher in patients with chemotherapy resistance than that of patients with chemotherapy sensitivity, H19 expression levels were higher in patients with breast cancer than that of their parental. (b) qPCR analysis showed that PARP1 expression levels were lower in patients with chemotherapy resistance than that of patients with chemotherapy sensitivity, PARP1 expression levels were lower in patients with breast cancer than that of their parental. *P < 0.01,**P < 0.001

To assess whether H19 has a role in breast cancer resistance to chemotherapy, qRT-PCR was used to analyze H19 expression in breast cancer patients. A total of 25 specimens from patients with chemotherapy sensitivity and 38 specimens from patients with chemotherapy resistance were included in this study (Table 1). Our results showed chemotherapy-resistant breast cancer tissue specimens exhibited generally higher *H19* levels compared with chemotherapy-sensitive tissues (Figure 1(a)). In addition, H19 levels were significantly upregulated in both chemotherapy-sensitive and chemotherapy-resistant cancer tissues relative to their adjacent normal tissues (Figure 1(a)).

**Table 1.** Clinical information of the 63 patients included in this study

	Doxorubicin sensitivity		Doxorubicin resistance	
Characteristics	Number (%)		Number (%)	
**Age 54 (23–69) years**	25		38	
<35	7	28	7	18.4
35-55	12	48	10	26.3
>55	6	24	21	55.3
**Histology**				
Infiltrating ductal carcinoma	12	48	10	26.3
Infiltrating (mixed) carcinoma	8	32	19	50
Others	5	20	9	23.7
**TNM stage**				
II	7	28	7	18.4
IIIA	11	44	9	23.7
IIIB-IV	7	28	22	58
**ER status**				
Negative	15	60	21	55.2
Positive	10	40	17	44.8
**PR status**				
Negative	18	72	23	60.5
Positive	7	28	15	39.5
**Her2 status**				
Negative	11	44	22	57.9
Positive	14	56	16	42.1

### PARP1 downexpression in breast cancer tissue correlates with chemoresistance

In addition, we detected the PARP1 mRNA levels in the adjacent normal tissues of above breast cancer tissues. As shown in Figure 1(b), PARP1 mRNA levels were significantly downregulated in both chemotherapy-sensitive and chemotherapy-resistant cancer tissues relative to their adjacent normal tissues.

### H19 is overexpressed and PARP1 is downexpressed in the Dox-resistant MCF-7 cells

**Figure 2.**
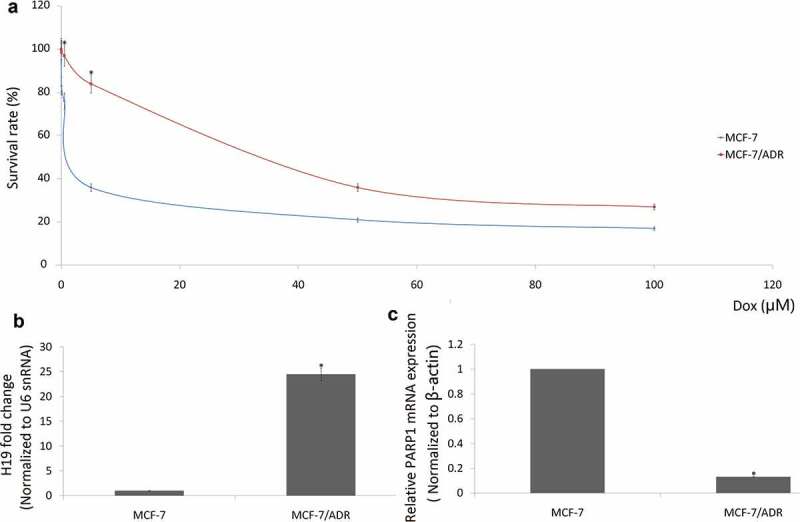
Overexpression of H19 enhances breast cancer resistance to Dox. (a) The IC50 values of MCF-7/Dox cells were higher than that of their parental MCF-7 cells. (b) qRT-PCR analysis of relative H19 expression in MCF-7/Dox cells and MCF-7 cells; (c), qRT-PCR analysis of relative PARP1 expression in MCF-7/Dox cells and MCF-7 cells. *P < 0.01

MCF-7 cells were continuously exposed to increasing concentrations of Doxorubicin (Dox) for 6 months. As shown in Figure 2(a), the IC50 of MCF-7 in Dox was 1.2 μM and 29.6 μM in the established MCF-7/Dox cells.

Consistent with the results from breast cancer tissues, high mRNA levels of H19 were found in doxorubicin-resistant MCF-7/Dox cells compared with the doxorubicin-sensitive MCF-7 cells (Figure 2(b)). On the contrary, low mRNA levels of PARP1 were found in doxorubicin-resistant MCF-7/Dox cells compared with the doxorubicin-sensitive MCF-7 cells (Figure 2(c)).

### H19 overexpression induces the chemoresistance of MCF-7 cells

**Figure 3.**
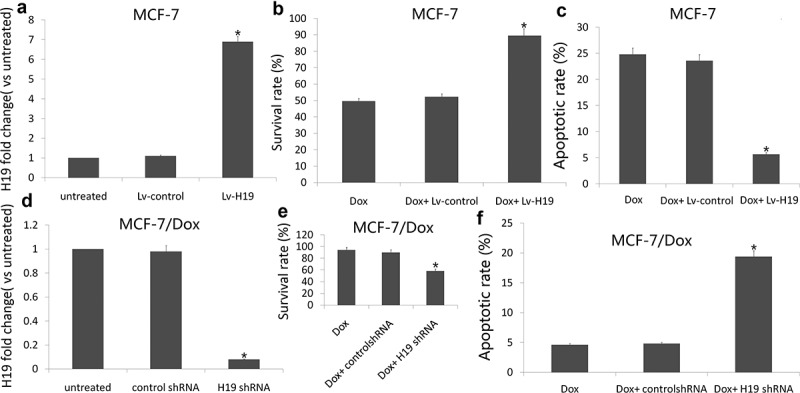
Targeting H19 enhances breast cancer sensitivity to Dox

We further investigated whether inhibiting or increasing H19 expression could modulate cell survival and the sensitivity of MCF-7 and MCF-7/Dox cells to Dox, which is currently used for the treatment of breast cancer. Following transfection of an pCMV6-H19 into MCF-7, we treated the cells with IC50 of Dox 1.2 uM for 72 h. Introduction of H19 notably increased H19 expression (Figure 3(a)) and reduced Dox (1.2 uM) induced cell apoptosis and increased cell viability (Figure 3(b,c)). In addition, following transfection of an **H19 siRNA** into MCF-7/Dox cells, we treated the cells with IC50 of Dox 1.2 uM for 72 h. Introduction of H19 siRNA notably decreased H19 expression (Figure 3(d)) and increased Dox (1.2 uM) induced cell apoptosis and decreased cell viability (Figure 3(e,f)).

MCF-7 cells were transfected with pCMV6 or pCMV6-H19, after 24 h, cells were treated with indicated 1.2 μM Dox for additional 72 h. (a) qRT-PCR analysis of relative H19 expression in MCF-7 cells; (b) Cell viability was detected by MTT assay; (c) Cell apoptosis was assessed by Annexin-V-FITC/PI staining assay by flow cytometry. MCF-7/Dox cells were transfected with NC siRNA or H19 siRNA, after 8 h, cells were treated with indicated 1.2 μM Dox for additional 72 h. (d) qRT-PCR analysis of relative H19 expression in MCF-7 cells; (e) Cell viability was detected by MTT assay; (f) Cell apoptosis was assessed by Annexin-V-FITC/PI staining assay by flow cytometry. *P < 0.01.

### Targeting H19 sensitizes breast cancer cells to doxorubicin through upregulating PARP1

**Figure 4.**
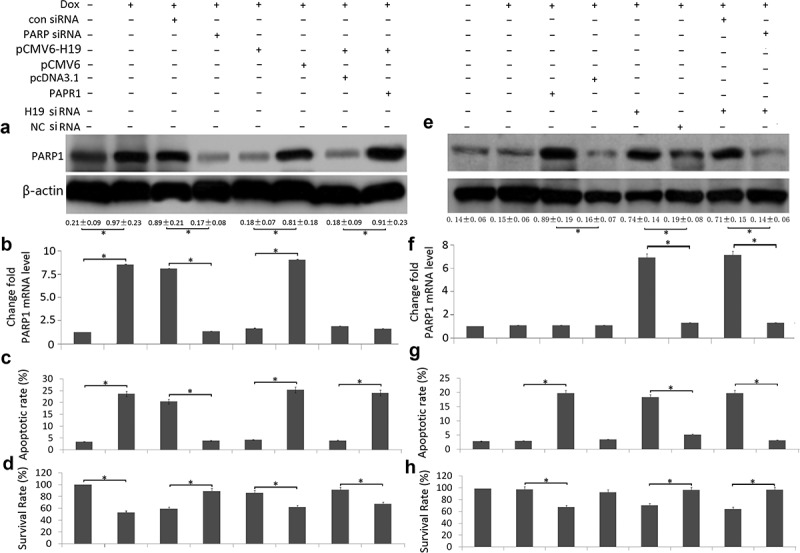
Targeting H19 reduced cell viability and induction of apoptosis by PARP1 upregulation in Dox-treated breast cancer cells

MCF-7 cells were treated with Dox (1.2 uM) for 72 h, PARP1 mRNA and protein was significantly upregulated compared to the controls (Figure 4(a,b)). After MCF-7 cells were transfected with PARP1 siRNA, Dox (1.2 uM) induced cell apoptosis was significantly decreased and cell proliferation was significantly increased, followed by decreased PARP1 mRNA and protein expression (Figure 4(c,d)).

MCF-7 cells were transfected or co-transfected with PARP siRNA, pCMV6-H19, pCMV6 – PARP1 and its controls and treated with Dox (1.2 uM) for 72 h. MCF-7/Dox cells were transfected or co-transfected with PARP siRNA, H19 siRNA, pCMV6-H19 and its controls and treated with Dox (1.2 μM) for 72 h. (a,e) PARP1 protein expression was analyzed by western blot assay; (b,f) PARP1 mRNA expression was analyzed by qRT-PCR assay; (c,g) Cell viability was detected by MTT assay; (d,h) Cell apoptosis was assessed by Annexin-V-FITC/PI staining assay by flow cytometry. *P < 0.01.

MCF-7 cells were treated with Dox (1.2 uM) for 72 h, H19 mRNA was significantly downregulated compared to the controls. After MCF-7 cells were transfected with **pCMV6-H19**, Dox-induced PARP1 mRNA and protein expression was decreased compared to the controls (Figure 4(a,b)), followed by decreased cell apoptosis and increased cell viability (Figure 4(c,d)). When MCF-7 cells were co-transfected with pCMV6-H19 and pCMV6-PARP1, PARP1 protein expression was increased (Figure 4(d)), Dox-induced cell apoptosis was increased and cell viability was decreased (Figure 4(c,d)).

MCF-7/Dox cells were treated with Dox (1.2 uM) for 72 h, PARP1 mRNA and protein did not change significantly compared to the controls (Figure 4(e,f)). After MCF-7/Dox cells were transfected with **pCMV6-**PARP1, PARP1 protein expression was upregulated (Figure 4(e)), Dox (1.2 uM) induced cell apoptosis was significantly increased and cell proliferation was significantly decreased compared to the controls (Figure 4(g,h)).

MCF-7/Dox cells were transfected with H19 siRNA, PARP1 mRNA and protein expression was upregulated compared to the control shRNA (Figure 4(e,f)). When MCF-7/Dox cells were co-transfected with H19 siRNA and PARP1 siRNA, PARP1 protein expression was decreased (Figure 4(e,f)), Dox-induced cell apoptosis was decreased and cell viability was increased (Figure 4(g,h)).

### Targeting H19 expression sensitizes breast cancer cells to Dox by targeting PARP1 in vivo

**Figure 5.**
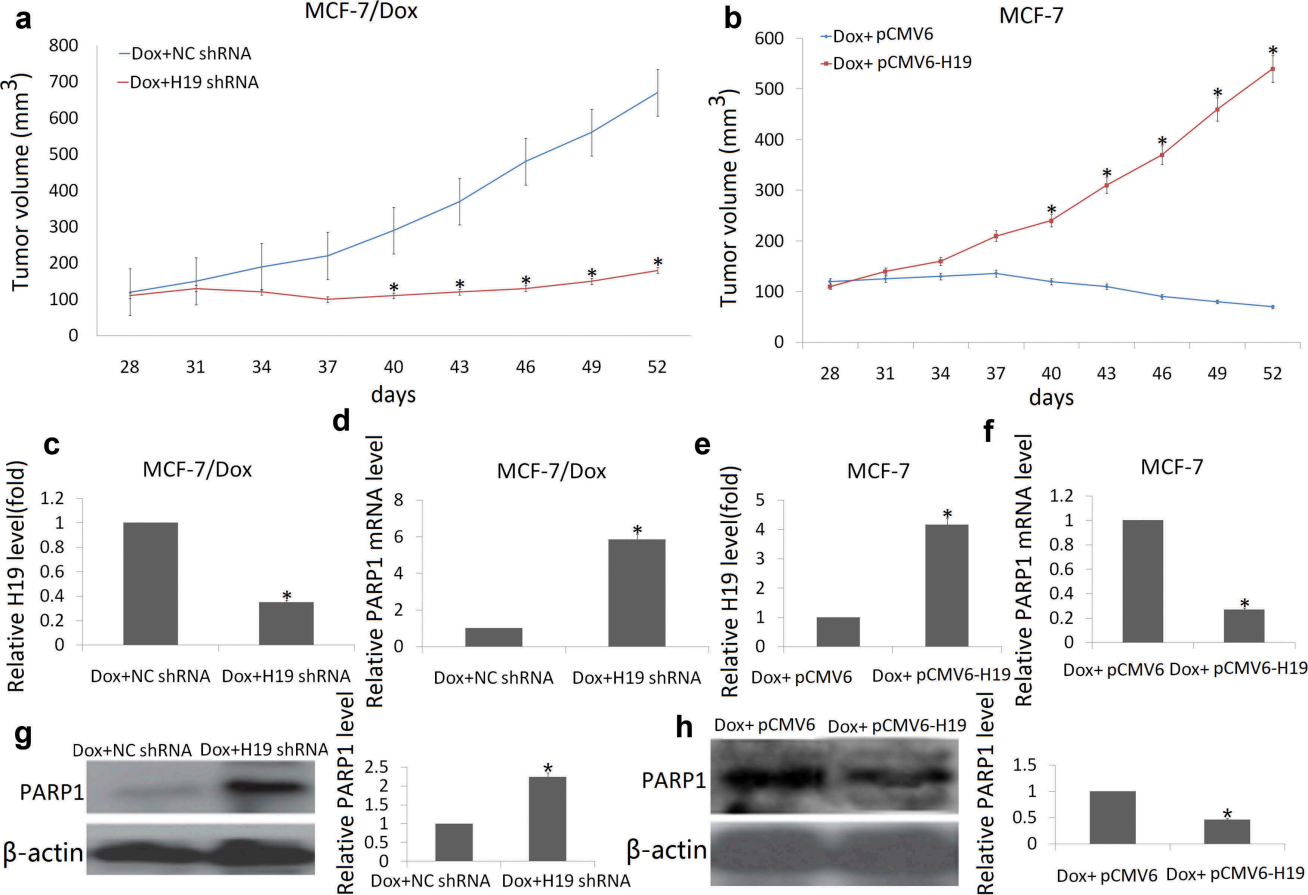
Targeting H19 decrease resistance of breast tumor to Dox in xenograft tumor models

MCF-7/Dox cells stably expressing a control vector (Lv-NC shRNA) or Lv-H19 shRNA were subcutaneously injected into the right fat pad of nude mice. The tumor volume was monitored every three days. In pre-experiment, NC shRNA or H19 shRNA alone did not significantly affect tumor (Data no shown). However, the tumor volume of H19 shRNA/Dox group was smaller than that of NC shRNA/Dox group, and significant difference in the tumor volume was observed between the two group (Figure 5(a)), suggesting that targeting H19 expression sensitizes breast cancer cells to Dox *in vivo*.

(a) MCF-7/Dox cells stably expressing H19 shRNA or NC shRNA were injected into nude mice. (b) MCF-7 cells stably expressing pCMV6-H19 or pCMV6 were injected into nude mice. When the tumor volume reached 100 mm^3^ (4 weeks), the mice (*n* = 8 per group) were, respectively, treated with PBS (0.1 mL, tail i.v. injection), Dox (0.1 mL, 10 mg/kg, tail i.v. injection, 4 times/week) and housed for another 24 consecutive days. Tumor volume was measured once per three days by using calipers (as indicated at each time point) for 24 days. (c, e) H19 mRNA expression was analyzed by qRT-PCR assay; (d,f) PARP1 mRNA expression was analyzed by qRT-PCR assay; (g,h) PARP1 protein expression was analyzed by western blot assay. *P < 0.01.

MCF-7 cells stably expressing a control vector (pCMV6) or pCMV6-H19 were subcutaneously injected into the right fat pad of nude mice. In pre-experiment, pCMV6 or pCMV6-H19 alone did not significantly affect tumor (Data no shown). However, the tumor volume of pCMV6-H19/Dox group was smaller than that of pCMV6/Dox group, and significant difference in the tumor volume was observed between the two group (Figure 5(b)), suggesting that enforced H19 was resistant to Dox breast cancer cells *in vivo*.

The mice were sacrificed at 24 days after Dox treatment, and their tumors were harvested. RNA and protein were extracted from the tumor tissues. The qRT-PCR data showed that the H19 levels (Figure 5(c)) were significantly decreased and PARP1 mRNA (Figure 5(d)) were significantly increased in the H19 shRNA groups; On the contrary, the H19 levels (Figure 5(e)) were significantly increased and PARP1 mRNA (figure 5(f)) were significantly decreased in the pCMV6-H19 groups. Western blot data showed that PARP1 protein expression was significantly increased in the H19 shRNA groups (Figure 5(g)) and significantly decreased in the pCMV6-H19 groups compared to the controls (Figure 5(h)).

## Discussion

Drug resistance in cancer patients is a serious clinical problem resulting in treatment failure. The main molecular pathological mechanisms responsible for drug resistance are the dysregulated expression of the drug transporters responsible for anti-cancer drug efflux, and the aberrant activation of anti-apoptotic, cell-survival and oncogenic pathways. During breast tumorigenesis, many miRNAs are aberrantly regulated to promote tumor cell survival [23–25]. Additionally, several miRNAs have been identified to target drug transporter genes in breast cancer cells. For instance, miR-451 and miR-326 are required for the chemosensitivity of breast cancer cells to doxorubicin via direct targeting of ABC family transporter genes ABCB1 and ABCC1 (MRP1), respectively [26,27].

Long noncoding RNAs (lncRNAs) of >200 nucleotides in length have recently emerged as key regulators of developmental processes, including mammary gland development [5]. lncRNA dysregulation has also been implicated in the development of various cancers, including breast cancer [7,8,10]. In breast cancer, aberrant lncRNA expression has been implicated to affect the response to various treatments, including chemotherapy, anti-endocrine therapies, targeted therapies, and radiotherapy [28,29]. In cancer, H19 is frequently overexpressed and it is associated with many aspects of cancer development, including breast cancer [9]. Zhang et al. demonstrated that the expression of H19 was significantly increased in breast cancer biopsies and plasma compared with healthy controls [30], plasma H19 levels were significantly correlated with progesterone and chemotherapy response [11].

In the present study, H19 expression was significantly upregulated in breast cancer tissues compared with normal breast tissues. Furthermore, H19 was significantly increased in chemoresistant breast cancer tissues and Doxorubicin-reaiatant MCF-7/Dox cell lines compared with the chemosensitive breast cancer tissues Doxorubicin-sensitive MCF-7 cell lines. These data indicated that H19 overexpression was related with chemoresistance in breast cancer.

A previous study speculated that the overexpression of H19 in breast cancer cells lines facilitates cell cycle transition G1/S while downregulation of H19 by RNA interference impedes S-phase entry and proliferation [31]. Recently H19 has been shown to be upregulated in paclitaxel-resistant breast cancer cells, and knockdown of H19 might restore chemo-sensitivity in paclitaxel-resistant breast cancer cells [15]. One of the most frequently used chemotherapeutics in breast cancer therapy is doxorubicin (Dox). Our *in vitro* and *in vivo* data suggested that H19 plays a role in the regulation of Dox-induced cell apoptosis in breast cancer. H19 downexpression increased Dox-induced cell apoptosis and enhanced the Dox response in the Dox-resistant MCF-7/Dox breast cancer. On the contrary, H19 downexpression decreased Dox-induced cell apoptosis and inhibited the Dox response in the Dox-sensitive MCF-7 breast cancer cells. These data indicated that targeting H19 could restore the DOX sensitivity in Dox-resistant breast cancer.

Recent clinical data confirmed the early in vitro studies and suggest that PARP-1 inhibitors could be used not only as chemosensitizers but as well as single agents to selective kill tumors with defective DNA repair by homologous recombination. For example, overexpression of miR-335 decreased the expression of PARP-1 expression, which was contributed to chemo-radiotherapy resistance in SCLC cells [32]. However, PARP1 has been shown to increase the antitumor activity of temozolomide and topotecan in preclinical studies, including models of pediatric cancers [33]. In the present study, PARP-1 expression was significantly downregulated in breast cancer tissues. Furthermore, PARP-1 was significantly increased in chemosensitive breast cancer tissues and Doxorubicin- chemosensitive MCF-7 cell. These data indicated that PARP-1 downexpression was related with chemoresistance in breast cancer. In our present study, PARP-1 expression is dramatically decreased and H19 expression is dramatically increased when MCF-7 cells are induced to acquire Dox resistance (MCF-7/Dox) in culture. When the H19 was knockdown in the MCF-7/Dox cells, PARP-1 expression was upregulated. Targeting H19 restored the sensitivity of MCF-7/Dox cells to Dox. However, the chemosensitivity to Dox was reversed in MCF-7/Dox cells when PARP-1 expression was blocked. In addition, H19 overexpression decreased Dox-induced PARP-1 expression and increased the acquired Dox resistance in Dox-sensitive MCF-7 cells. The role of H19 overexpression in acquired Dox resistance can be reversed by PARP-1 re-expression in the Dox-sensitive MCF-7 cells. Furthermore, blocking the action of H19 alone is sufficient to restore PARP-1 expression by Dox in the resistant cells, and is capable of sensitizing the resistant cells to Dox in vivo, and *vice versa*.

## Conclusion

We identified the function and target of H19 in regulating chemotherapy response both in cell lines and in clinical samples, providing evidence that H19 targets PARP-1 to mediate the chemotherapeutic resistance of breast cancer cells. We extended the current knowledge by highlighting the role of H19 in the chemoresistance of breast cancer for the first time. Although chemotherapy is the backbone of systemic treatment for most malignancies, its efficacy is hindered by the development of drug resistance. Therefore, targeting the mechanisms involved in the chemoresistance of breast cancer will improve treatment efficacy. It is logical to predict that H19 could be a potential biomarker and that the inhibition ofH19 may be a candidate strategy for new breast cancer therapies.

## Highlights

1. H19 overexpression is related with chemoresistance in breast cancer tissues and doxorubicin-resistant cells.
2. Targeting H19 enhanced the sensitivity to doxorubicin both *in vitro* and *in vivo* by PARP1 upregulation.
3. H19 overexpression recapitulates doxorubicin resistance by PARP1 downregulation.
